# In Vivo Characterization of the Anti-Glutathione S-Transferase Antibody Using an In Vitro Mite Feeding Model

**DOI:** 10.3390/vaccines12020148

**Published:** 2024-01-30

**Authors:** Shwe Yee Win, Hikari Seo, Fumiya Horio, Sotaro Fujisawa, Jumpei Sato, Yoshinosuke Motai, Takumi Sato, Eiji Oishi, Akira Taneno, Lat Lat Htun, Saw Bawm, Tomohiro Okagawa, Naoya Maekawa, Satoru Konnai, Kazuhiko Ohashi, Shiro Murata

**Affiliations:** 1 Laboratory of Infectious Diseases, Department of Disease Control, Faculty of Veterinary Medicine, Hokkaido University, Kita-18, Nishi-9, Kita-ku, Sapporo 060-0818, Japan; 2 Vaxxinova Japan K.K., 1-24-8 Hamamatsucho, Minato-ku, Tokyo 105-0013, Japan; 3 Department of Pharmacology and Parasitology, University of Veterinary Science, Yezin, Nay Pyi Taw 15013, Myanmar; 4 Department of Livestock and Aquaculture Research, Ministry of Agriculture, Livestock and Irrigation, Nay Pyi Taw 15013, Myanmar; 5 Department of Advanced Pharmaceutics, Faculty of Veterinary Medicine, Hokkaido University, Kita-18, Nishi-9, Kita-ku, Sapporo 060-0818, Japan; 6 Institute for Vaccine Research and Development (GU-IVReD), Hokkaido University, Sapporo 060-0818, Japan; 7 International Affairs Office, Faculty of Veterinary Medicine, Hokkaido University, Kita-18, Nishi-9, Kita-ku, Sapporo 060-0818, Japan

**Keywords:** poultry red mite, tropical fowl mite, northern fowl mite, glutathione S-transferase, vaccine

## Abstract

Poultry red mites (*Dermanyssus gallinae*, PRMs), tropical fowl mites (*Ornithonyssus bursa*, TFMs), and northern fowl mites (*O. sylviarum,* NFMs) are blood-feeding pests that debilitate poultry worldwide. Glutathione S-transferase (GST) plays an important role in the detoxification and drug metabolism of mites. However, research on avian mite GSTs as vaccine antigens is still lacking. Therefore, we aimed to evaluate the potential of avian mite GSTs for vaccine development. We identified GST genes from TFMs and NFMs. We prepared recombinant GST (rGST) from TFMs, NFMs, and PRMs, and assessed their protein functions. Moreover, we evaluated the cross-reactivity and acaricidal effect of immune plasma against each rGST on TFMs, NFMs, and PRMs. The deduced amino acid sequences of GSTs from TFMs and NFMs were 80% similar to those of the PRMs. The rGSTs exhibited catalytic activity in conjugating glutathione to the 1-chloro-2,4-dinitrobenzene substrate. Immune plasma against each rGST showed cross-reactivity with rGST from different mite species. Moreover, the survival rate of PRMs fed with immune plasma against the rGST of TFMs and NFMs was significantly lower than that of the control plasma. These results demonstrate the potential application of GST as an antigen for the development of a broad-spectrum vaccine against avian mites.

## 1. Introduction

Hematophagous avian mites, including poultry red (*Dermanyssus gallinae*, PRMs), tropical fowl (*Ornithonyssus bursa*, TFMs), and northern fowl mites (*Ornithonyssus sylviarum*, NFMs), parasitize wild birds and poultry [1,2,3,4]. Avian mites are genetically similar and non-uniformly distributed worldwide [4]. Avian mites have a short life cycle (one week) from larvae to adults. Host blood meals are necessary for mite development [5,6,7]. PRMs suck blood during the protonymph, deutonymph, and adult stages, whereas protonymphs and adults of TFMs/NFMs require blood meals. PRMs suck the blood at night, leave their hosts, and hide in farm equipment and cracks in poultry houses during the daytime, whereas TFMs and NFMs are permanently on-host parasites [6,7]. Heavy infestations of PRMs and TFMs/NFMs cause chickens to lose 3% of their whole blood per night [6] and 6% of their whole blood per day [7], respectively. Blood feeding and prompt reproductive dynamics affect poultry welfare and production in farms and decrease feed conversion efficacy. Additionally, PRMs can act as vectors for several zoonotic avian pathogens, *Salmonella Enteritidis* (Salmonellosis or foodborne illness) [8], *Erysipleothrix rhusipathiae* (Erysipelas) [9], *Escherichia coli* (gastroenteritis, urinary tract infections, etc., the pathogenicity depends on serotypes) [10], *Chlamydia psittaci* (Chlamydiosis) [11], *Pasteurella multocida* (Pasteurellosis or fowl cholera), *Coxiella burnetti* (Q fever), *Listeria monocytogens* (Listeriosis) [12], and influenza type A virus [13]. Workers at farms contaminated with avian mites are at risk for gamasoidosisis (dermatitis) [14]. In addition, urban birds such as pigeons are factors for gamasoidosisis (dermatitis) cases in humans because they often build their nests near human facilities [15]. In the European Union, 90% of the poultry industry is affected by PRMs. The decreased production and PRM control cost more than €231 million per year [16]. Infestation with NFMs can reduce egg production (2.1–4.0%), individual egg weight (0.5–2.2%), and feed conversion efficiency (5.7%), which is equivalent to a profit reduction of $0.07–0.10 per hen for a 10-week period [17].

Chemical methods are commonly used to control avian mites in the poultry industry. However, acaricides have become ineffective against avian mites due to the selection of resistant mites by long-term repeated use of acaricides on farms or the sharing of resistant genes that have similar mechanisms against different acaricides [18,19]. In addition, chemical residues in meat, eggs, and environmental pollution are major concerns for humans [20]. To overcome these problems, alternative control approaches, including vaccines against PRMs, are being investigated [21,22,23,24,25,26,27,28,29,30,31,32,33,34,35,36]. Our research group targeted antigens with cross-protective efficacy against avian mites [37,38]. Thus, vaccines against avian mites may become a cost-effective strategy to control avian mites in the poultry industry. Several PRM molecules have been reported as vaccine antigen candidates, and some have been evaluated as antigens that have the potential for the development of universal vaccines. However, for the commercial application of vaccines on poultry farms, the combined use of antigens as cocktail vaccines is required to enhance efficacy. According to the principle of anti-tick cocktail vaccines, the combination of several antigens could induce higher protective efficacies than the use of a single antigen, because it can inhibit multiple physiological functions [39]. A combination of antigens that have the potential to protect against a particular tick species could be a good formulation for cocktail vaccines because it expands the protection range of the hosts, increases efficacies against the different developmental stages, and interferes with biological parameters and pathogen transmission. However, the phenomenon of antigenic competition via inter- or intra-molecular competitive mechanisms still remains controversial [39]. Therefore, further investigation for antigen candidates could develop an effective vaccine against mites.

Glutathione S-transferases (GSTs) are a family of intracellular enzymes important for the protection against oxidative stress, aging, cancer, and metabolic detoxification of endogenous and xenobiotic compounds such as drugs, herbicides, and insecticides [40,41]. GSTs are present in various tissues and catalyze a variety of compounds in the presence of glutathione (GSH), resulting in fewer harmful compounds [42]. GSTs from several species of parasites and pests, such as house dust mites, parasitic nematodes, and cockroaches, cause allergenic reactions in vertebrates and humans [43,44]. Alpha, mu, theta, and sigma classes of GSTs have been identified in chickens, and these heterologously expressed proteins have unique properties as avian GSTs [45]. The efficacy of acaricides depends on the detoxification mechanism of GST in ticks [46,47]. Given the significant roles of GSTs in detoxification and drug metabolism, they are of great interest as novel control strategies against several ecto- and endoparasites [48,49,50]. The GSTs are potential vaccine antigen candidates for anti-tick vaccines [51,52]. Moreover, specific B-cell epitope-based GST vaccines have been used to assess the cross-protective potential against tick species [53]. In PRMs, two classes of GSTs, delta and mu, have been identified, and their potential for acaricide detoxification has been demonstrated [54]. However, research on avian mite GSTs as vaccine antigens is still lacking.

Therefore, this study aimed to evaluate the potential of avian mite GSTs for vaccine development. Our study found that the GSTs of PRMs, TFMs, and NFMs are highly conserved. In addition, rGSTs exhibit enzymatic activity in the conjugation of GSH to substrates, and their immunization successfully produces specific antibodies in chickens. Furthermore, immune plasma against rGST from PRMs, TFMs, and NFMs shows cross-reactivity with rGSTs from different avian mites and acaricidal effects on all avian mites.

## 2. Materials and Methods

### 2.1. Ethic Statement

The chickens used in this study were housed in the animal facility at the Faculty of Veterinary Medicine, Hokkaido University, with the approval of the Institutional Animal Care and Use Committee of Hokkaido University (approval number 22-0051). The animal experiments were performed in accordance with the regulations fully accredited by the Association for Assessment and Accreditation of Laboratory Animal Care International.

### 2.2. Mite Samples and RNA Extraction

PRM samples were obtained from egg-laying farms contaminated with PRMs in Japan. PRMs were collected in a TubeSpin bioreactor 600 bottle (TPP Techno Plastic Products AG, Trasadingen, Switzerland) and transferred to the laboratory at 4 °C. For further analysis, PRMs were stored at 25 °C and 70% humidity for a week without blood feeding to design starved PRMs and produce newborn nymphs. The mixed stages of starved PRMs were stored at 5 °C. We used RNA samples from TFMs and NFMs that were morphologically and genetically characterized in a previous study [55]. The total RNA from each mite species was extracted using the RNeasy RNA isolation kit (Qiagen, Germantown, MD, USA) according to the manufacturer’s protocol. One microgram of RNA was used to synthesize complementary DNA (cDNA) as previously described [37,38].

### 2.3. Amplification of GST

The GST genes from TFMs and NFMs were amplified using the primers listed in Supplementary Table S1. Primers for the partial gene amplification of each GST were designed based on the sequences of PRMs (*D. gallinae*; accession no. KR337505.1), *Varroa jacobsoni* (accession no. XM022854296.1), and *V. destructor* (accession no. XM022792712.1). Partial gene segments were cloned into the pMD20 vector (Takara Bio Inc., Shiga, Japan), and sequence analysis was performed using a Beckman CEQ GeXP automated sequencer (Beckman Coulter Inc., Brea, CA, USA). For the identification of the open reading frames (ORFs) of GSTs, we conducted rapid amplification of cDNA ends (Invitrogen, Carlsbad, CA, USA) analysis to amplify the unknown region of 3′ and 5′ sites. Primers for this analysis were designed based on the partial nucleotide sequences (Supplementary Table S1). The ORF of PRM GSTs from Japan was confirmed using primers designed based on the ORF regions of GST (Supplementary Table S1). The fragments were separated using 1.5% agarose gel electrophoresis and purified. The purified DNA products were cloned into pGEMT easy vector (Promega, Madison, WI, USA) and transformed into competent DH5α *Escherichia coli* cells (Takara Bio Inc.).

### 2.4. Genetic Characterization of GSTs from Avian Mites

The ORF sequences of the GSTs of PRMs, TFMs, and NFMs were determined and assigned accession nos. LC776937, LC776938, and LC776939, respectively. In addition, we used PRM GST (accession no. KR337505.1) as reported in GenBank. A homology search for GSTs was performed using the Basic Local Alignment Search Tool of the National Center for Biotechnology Information (NCBI). The InterPro95.0 (https://www.ebi.ac.uk/interpro/, accessed on 19 September 2023) and Conserved Domain search programs (https://www.ncbi.nlm.nih.gov/Structure/cdd/wrpsb.cgi, accessed on 19 September 2023) of NCBI were used to identify the functional domains and active residues from the translated sequences of each GST from avian mites. For the 3D structural analysis of GSTs, we used the SWISS-MODEL (https://swissmodel.expasy.org/, accessed on 20 September 2023), an automated homology modeling server. The protein structures of GSTs from TFMs and NFMs were built by using the alpha-fold monomeric structure of PRM GST (A0A0H4FNI7) as a template. The structures of GSTs from the PRMs, TFMs, and NFMs were manipulated using the PyMOL program (version 2.5.5). MEGA software (version X) was used to construct the phylogenetic tree [56]. The maximum likelihood phylogeny was built with 1000 bootstrap values, a discrete gamma distribution (+G), and assuming that a certain fraction of sites were evolutionarily invariable (+I) to improve tree topology.

### 2.5. Generation of rGSTs

The expression and purification of recombinant proteins were performed as described previously [27,28]. Briefly, the rGST proteins from PRMs, TFMs, and NFMs were expressed as fusion proteins with a histidine tag using an *E. coli* expression system and termed rGST PRM, rGST TFM, and rGST NFM, respectively. The coding regions of GSTs from PRMs, TFMs, and NFMs were amplified using specific primers including NdeI and XhoI restriction sites (Supplementary Table S1). The products were cloned into the NdeI and XhoI restriction sites of the pET19b vector (Merck & Co., Inc., Rahway, NJ, USA) and transformed into *E. coli* (DE3) [Merck]. The inclusion body fractions containing the target proteins were separated using BugBuster solution (Merck) and solubilized in buffer containing 0.3% N-lauroylsarcosine and 50 mM N-cyclohexyl-3-aminopropanesulfonicacid (CAPS; Merck; pH 11.0). The rGST proteins were purified using the Ni Sepharose™ 6 Fast Flow resin (GE Healthcare, Chicago, IL, USA). The rGST proteins were eluted with 0.3% N-lauroylsarcosine and 50 mM CAPS (pH 11.0) buffer containing 250 mM imidazole (Nacalai Tesque, Tokyo, Japan). The refolding dialysis was performed against 10 mM Tris-HCL (pH 8.5) buffer containing 0.1 mM DL-Dithiothreitol (Merck) at 4 °C overnight. The purity of rGSTs was confirmed by sodium dodecyl sulfate–polyacrylamide gel electrophoresis (SDS-PAGE) and western blotting. The concentration of rGSTs was measured using a Pierce™ Bicinchoninic Acid Protein Assay Kit (Thermo Fisher Scientific, Waltham, MA, USA) with bovine serum albumin (BSA) as a standard.

### 2.6. Enzyme Activity Assay

rGSTs of PRMs catalyze the conjugation of GSH to 1-chloro-2,4-dinitrobenzene (CDNB) substrate [44]. In this study, we measured enzyme activity at different concentrations of each rGST in the presence of a constant concentration of GSH and the CDNB substrate. Briefly, 20 µL of each rGST (1, 2, and 4 µg) in Tris/NaCl buffer (10 mM Tris, 0.5 M NaCl, pH 7.4) were placed in the 96-well microtiter plate in triplicate and the same amount of Tris/NaCl buffer was placed in the control wells. We dissolved 100 µL of 1 mM CDNB in substrate buffer (100 mM potassium dihydrogen phosphate, 1 mM EDTA, pH 6.5, and 2 mM GSH) and placed it in all the wells. Immediately, the absorbance at 340 nm was measured every minute using a microplate reader MTP 900 (Corona Electric Co., Ltd, Ibataki, Japan) for 20 min. To adjust the spontaneous hydrolysis of CDNB, the mean absorbance of the control wells was subtracted from that of the test wells. The specific enzyme activities (μmol/min/mg protein) at different concentrations of each rGST were calculated using the following formula [54].

(1) $$\frac{\left(At2-At1\right)\times 1000}{A\varepsilon \times \left(t2-t1\right)\times b\times m}\times Vtot$$

At1—initial time point

At2—final time point

t1—starting time (min)

t2—end time (min)

Aε—the molar extinction coefficient of CDNB (Aε = 9.6)

b—path length of the spectrophotometer (0.286 cm)

m—the quantity of rGST per well (mg)

Vtot—total volume per well (L).

### 2.7. Production of Anti-rGST Antibody

To produce specific antibodies against GSTs of PRMs, TFMs, and NFMs, four chickens per group were subcutaneously injected with 20 µg per 0.5 mL of each rGST emulsified with Freund’s incomplete adjuvant (FUJIFILM Wako Pure Chemical Corporation, Tokyo, Japan) at 3 and 7 weeks with 21-G needles. For the control group, we used phosphate buffer saline (PBS) with the same adjuvant. Three weeks after the second administration, chickens were euthanized by collecting heparinized whole blood from hearts of each immunized chicken separately, under deep general anesthesia with Isoflurane inhalation (Zoetis Japan, Tokyo, Japan), and approximately 25–30 mL of heparinized blood was obtained. The immune plasmas were isolated for further experimentation.

### 2.8. Enzyme-Linked Immunosorbent Assay (ELISA)

The antibody titers in the plasma isolated from each chicken were determined by using ELISA [27,28]. The rGST of PRMs, TFMs, and NFMs (100 ng/well) was coated onto a 96-well plate (Sumitomo Bakelite Co. Ltd., Tokyo, Japan) using a carbon-bicarbonate buffer (pH 9.8) at 4 °C overnight. After washing the plate three times with PBS containing 0.05% Tween 20 (PBS-T), the plate was blocked with 1% BSA at 37 °C for 1 h. The plates were then washed five times with PBS-T. A volume of 100 µL of diluted immune plasma (2000×, 4000× 8000×, 16,000×, 32,000×, and 64,000×) obtained from each chicken was used as the primary antibody to react with the antigen-coated plate. The plate was incubated at 25 °C for 1 h and washed five times with PBS-T. The reaction complex was labeled with a 10,000× dilution of goat anti-chicken IgY[IgG](H+L)-horseradish peroxidase (HRP) (Bethyl Laboratories, Inc., Montgomery, TX, USA) for 1 h at 37 °C. Thereafter, the plate was washed five times with PBS-T and incubated with 3, 3′, 5, 5′-tetramethylbenzidine one component HRP microwell substrate (Bethyl Laboratories, Inc., USA) at 25 °C for 20 min in the dark. Finally, the reaction was stopped by using 0.18 M H_2_SO_4_, and immediately, the absorbance was measured at 450 nm using an ELISA plate reader MTP 900 (Corona Electric Co., Ltd., Ibaraki, Japan). The absorbance values of the control plasma were below 0.42 for all GSTs at 1000× and 2000× dilution. Therefore, each dilution of plasma from immunized chickens was considered positive if the absorbance values were above 0.42.

### 2.9. Western Blotting

The production of specific antibodies against each rGST and the cross-reactivity of each anti-rGST antibody with rGSTs from different avian mites were confirmed by western blotting [27,28]. One microgram of each rGST was resolved by 13% SDS-PAGE and transferred to polyvinylidene difluoride membranes (Merck). The transferred membranes were blocked with 1% skim milk in PBS-T at 4 °C overnight and incubated with each anti-GST chicken plasma (1:1000) at 25 °C for 1 h. Membranes were washed with PBS-T three times and labeled with anti-chicken IgY peroxidase rabbit antibody (1:10,000) [Sigma-Aldrich, St. Louis, MO, USA] at 25 °C 1 h. Signal detection was performed by incubating the membrane with Immobilon Western Chemiluminescent HRP Substrate (Merck) for 5 min at 25 °C. To confirm His-tagged rGST purification, each rGST was incubated with HRP-conjugated anti-6-his, mouse monoclonal antibody (1:6000; Merck) and labeled with goat anti-mouse IgG antibody (1:10,000; Merck), as described above.

### 2.10. Assessment of Acaricidal Potential by Using Immune Plasma

To assess the acaricidal efficacy of rGSTs, PRMs were fed with immune plasma against each GST using artificial feeding devices (in vitro feeding assay) [27,28,57], and the mortality rate of blood-fed PRMs was monitored. In the present study, we were only able to use PRMs for these assays because of the limited availability of TFMs and NFMs in Japan. Fresh blood cells were centrifugally separated from blood obtained from healthy chickens housed at the Field Science Center for the Northern Biosphere, Hokkaido University. The plasma of healthy chickens was replaced with pooled plasma from each immunized or control group. To construct the devices for the artificial feedings, we cut 10 mL serological disposable pipets (Corning Inc., Corning, NY, USA) and assembled them across stretched Parafilm (Bemis Co., Inc., Neenah, WI, USA). Mixed stages of starved PRMs stored at 5 °C were kept at 25 °C one day before the assays and collected using 300 µL Universal Barrier Tips (Neptune Scientific, San Diego, CA, USA). Tips containing starved PRMs were inserted in the devices, and subsequently, the open ends of the pipettes were closed with rubber caps (Cytiva, Uppsala, Sweden). For air ventilation, 27-G needles were stabbed into the rubber caps. After placing the starved PRMs in the devices, the tops of the devices were filled with 400 µL of blood containing immune plasma, and blood feeding was performed in a dark and humid environment with modest shaking for 4 h at 40 °C. Blood-fed PRMs from each feeding group were collected with a Pasteur pipette 4 h after blood feeding. We previously demonstrated that a certain number of blood-fed PRMs could be obtained using this method [37,38]. The collected PRMs were maintained at 25 °C and 70% humidity to monitor their mortality. The mortalities of the blood-fed PRMs were recorded daily for seven days. Artificial feeding assays were conducted in duplicate. The number of PRMs monitored was as follows: Experiment 1: fed with immune plasma against rGST PRM: *n* = 153, rGST TFM: *n* = 113, rGST NFM: *n* = 148, control plasma: *n* = 149; Experiment 2: rGST PRM: *n* = 228, rGST TFM: *n* = 107, rGST NFM: *n* = 203, control plasma: *n* = 143. For each assay, the same batch of PRMs was used, with the same collection site and date, and transported and stored in a single tube.

### 2.11. Statistical Analysis

The EZR statistical software was used for statistical analysis [58]. Fisher’s exact test with odds ratios, 95% confidence intervals (CI), and Chi-square values were used to compare the daily mortality rate of PRMs between each immunized and control group. To compare the mortality of PRMs between the immunized and control groups for seven days after in vitro feeding, a log-rank test was performed and Kaplan–Meier survival curves were generated. The significant difference with the control group was set at *p* < 0.05 and *p* < 0.01 for the Fisher’s exact and log-rank tests, respectively.

## 3. Results

### 3.1. Characterization of GSTs from Avian Mites

The nucleotide sequence of PRM GST from Japan was 100% identical to the reported GST sequences of PRMs (accession no. KR337505). The nucleotide and deduced amino acid sequences of GSTs from TFMs and NFMs were 75 and 80% identical, respectively, to those of PRMs, and the homology of GSTs between TFMs and NFMs was 100% (Table 1).

**Figure 1:**
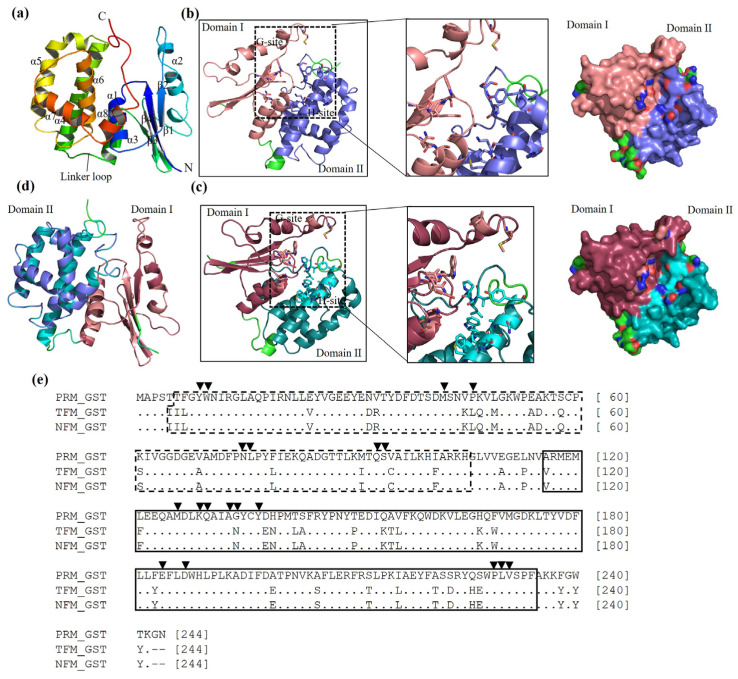
Characterization of glutathione S-transferases (GSTs) from avian mites. (**a**) Cartoon views of PRM GST labeled with the monomer structure (α1–α8, β1–β4, and linker loop). Cartoon views of GSTs from (**b**) poultry red mites (PRMs) and (**c**) tropical fowl mites (TFMs)/northern fowl mites (NFMs). The right panels indicate the surface structure of GSTs locating their binding sites. The monomer structure of GSTs contains N-terminal Domain I and C-terminal Domain II connected with a linker loop. The ligand-binding sites (G- and H-sites), for binding multiple substrates and catalytic function, are shown in stick format. Domains I and II are indicated by salmon and slate colors in PRMs and raspberry and teal colors in TFMs/NFMs. (**d**) The 3D structure alignment of GSTs from PRMs, TFMs, and NFMs. (**e**) Multiple sequence alignments of GSTs where the monomer structure, functional domains, and residues in binding sites are labeled. The structure of GST contains Domain I (dashed box) at positions of 6–105 in PRMs and 5–105 in TFMs and NFMs and Domain II (black box) at positions of 116–234 in PRMs, TFMs, and NFMs. The black arrowhead indicates the active residues of G- (Domain I) and H-sites (Domain II).

The monomeric structures of GSTs from PRMs, TFMs, and NFMs are shown in Figure 1a–d. The predicted monomer form of GSTs of PRMs, TFMs, and NFMs was composed of N terminal Domain I, containing 1–4 β strands and 1–4 α helices, and C terminal Domain II, containing 5–8 α helices. Domains I and II were connected via the linker loop region. As a general feature of GSTs, Domains I and II had representative active residues responsible for two ligand-binding sites, G- and H-sites, respectively. The G-site residues, Tyr9, Tyr10, Met42, Pro46 (Leu46 in GSTs of TFMs and NFMs), Asn75, Leu76, Gln93, and Ser94, and the H-site residues, Met126, Lys129, Gln130, Ala133, Gly134 (Asn134 in GSTs of TFMs and NFMs), Tyr137, Phe185, Trp188, Pro229, Leu230, and Val23, are supposed to bind multiple substrates and are capable of catalytic functions. As shown in the multiple sequence alignment, GSTs from TFMs and NFMs were conserved among PRMs (Figure 1d,e). In addition, the active residues in each domain were conserved among avian mites. Therefore, the GSTs of avian mites appear to have the capacity to perform similar functions.

**Figure 2:**
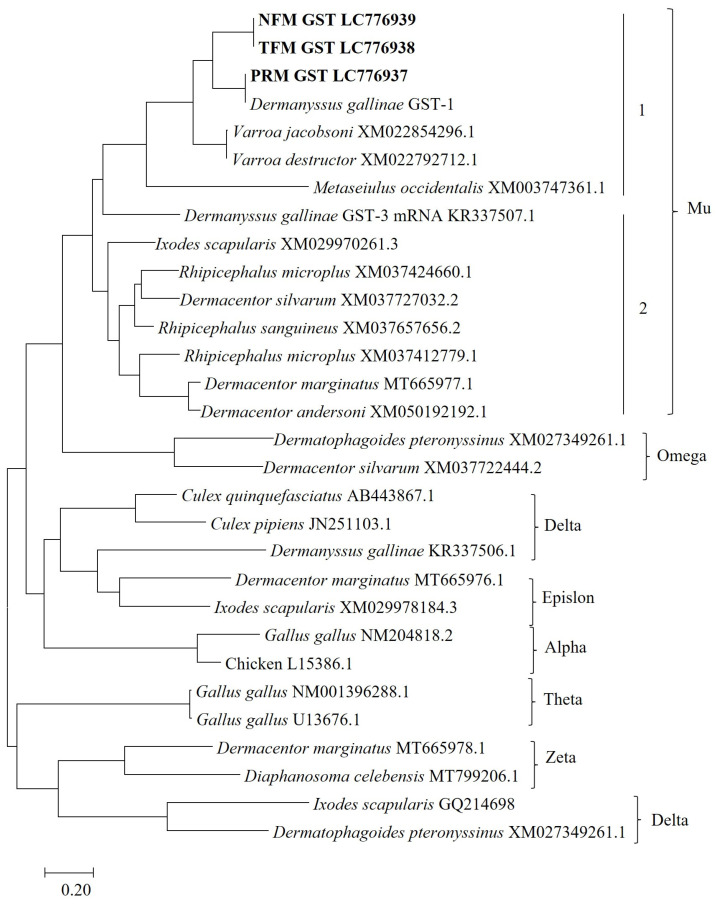
Phylogenetic analysis of GST nucleotide sequences derived from avian mites. The assigned GST classes are labeled. Subclusters 1 and 2 of the mu clade indicate the mu-class GST of mites and ticks, respectively. The GSTs of PRMs, TFMs, and NFMs are shown in bold.

The phylogenetic tree constructed using different classes of GSTs from arthropods, including other mites and ticks, are presented in Figure 2 and Supplementary Table S2. The tree was divided into seven clusters: mu, alpha, zeta, delta, omega, epsilon, and theta. The GSTs of avian mites were partitioned within the mu class. The mu clade was grouped into two subclades, subclades 1 and 2, representing the mu-class GST of mites and ticks, respectively. Therefore, the GSTs of avian mites seem to be similar to the mu-class GSTs of other mites and ticks.

### 3.2. Enzyme Activity of rGSTs from PRMs, TFMs, and NFMs

**Figure 3:**
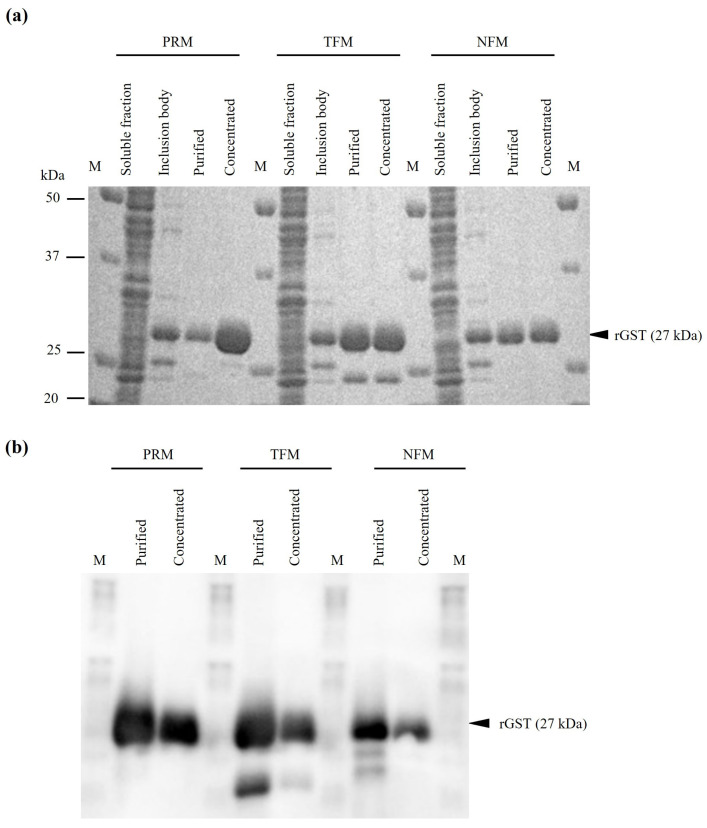
Generation of rGST proteins. The rGSTs were purified and designated as rGST PRM, rGST TFM, and rNFM NFM. The expressions of rGSTs were confirmed by (**a**) SDS-PAGE and (**b**) western blotting. M, Marker (Precision Plus Protein™ All Blue Prestained Protein Standards, Bio-Rad, Hercules, CA, USA).

To analyze the function of GSTs, we generated rGST proteins from PRMs, TFMs, and NFMs and designated them as rGST PRM, rGST TFM, and rGST NFM, respectively. Although the GST sequences of the TFMs and NFMs were 100% identical, we separately generated rGST proteins from the TFMs and NFMs to increase the reproducibility of subsequent experiments. As predicted, the purified proteins were expressed at 27 kDa (Figure 3a,b).

**Figure 4:**
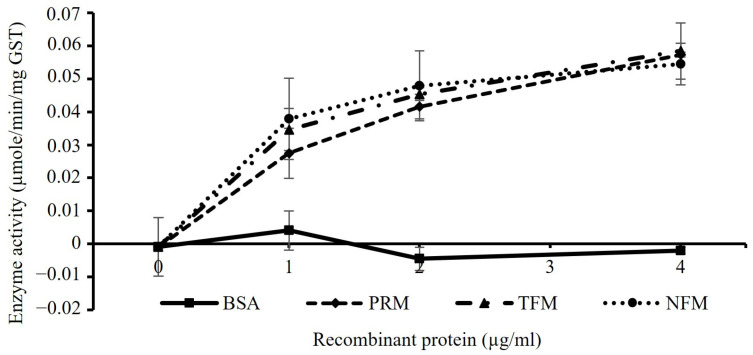
Enzyme activity of rGSTs. The colorimetric-based enzyme activities of rGSTs from PRMs, TFMs, and NFMs was conducted in triplicate. The specific enzyme activities (μmol/min/mg protein) upon the different concentrations of each rGST were calculated. The x-axis indicates the concentration of rGSTs used in these assays. The error bar indicates the standard deviations. BSA, bovine serum albumin.

The CDNB-based colorimetric assay revealed that the GSTs of the PRMs, TFMs, and NFMs were enzymatically active and capable of catalyzing the conjugation of GSH to CDNB. The enzymatic activity of each GST was plotted in a dose-dependent manner (Figure 4).

### 3.3. Production of Antibodies against rGSTs and Their Cross-Reactivities with rGSTs of Different Mites

**Figure 5:**
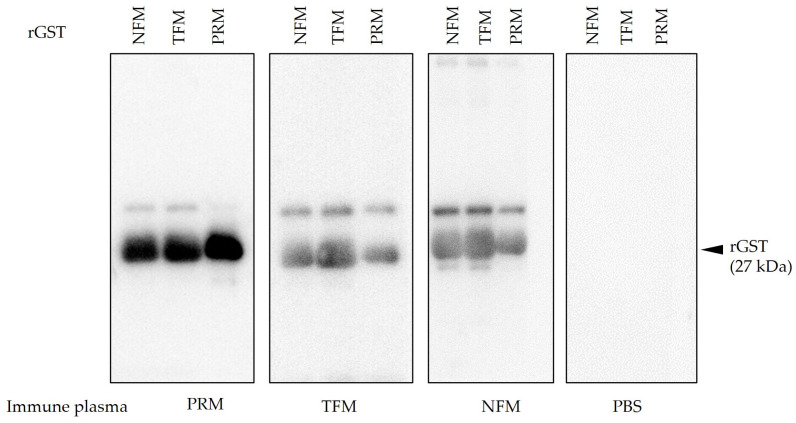
The production of antibodies specific to each rGST in the plasma from immunized chickens. The black arrowhead indicates the predicted molecular weight of rGSTs (27 kDa). PBS, phosphate buffer saline.

Chickens were immunized with each rGST and immune plasmas were isolated separately. Increased antibody production was observed in immunized groups. A high antibody titer of 32,000–64,000 was confirmed three weeks after the second immunization (Table 2). The presence of specific antibodies against each rGST was confirmed by western blotting. In addition, the immune plasma obtained from chickens immunized with each GST cross-reacted with rGSTs from different mite species, and vice versa (Figure 5). When the immune plasma against rGST PRM was reacted with rGSTs from TFMs and NFMs, a slight signal difference was observed, compared with that of rGST from PRMs. However, the signal intensities were not different when the plasmas obtained from chickens immunized with rGST TFM and NFM were reacted with rGSTs from different avian mites. This difference may be because some epitopes in the GSTs of TFM and NFM are slightly different from those of PRM. Another signal was found at higher molecular weights in each lane than the expected size. In addition, this signal was faint for the PRM immune plasma when tested against rGST PRM. This signal may be attributed to residual protein dimers or aggregates, but it is unclear why it was generated.

### 3.4. In Vitro Acaricidal Effect of Immune Plasma

**Figure 6:**
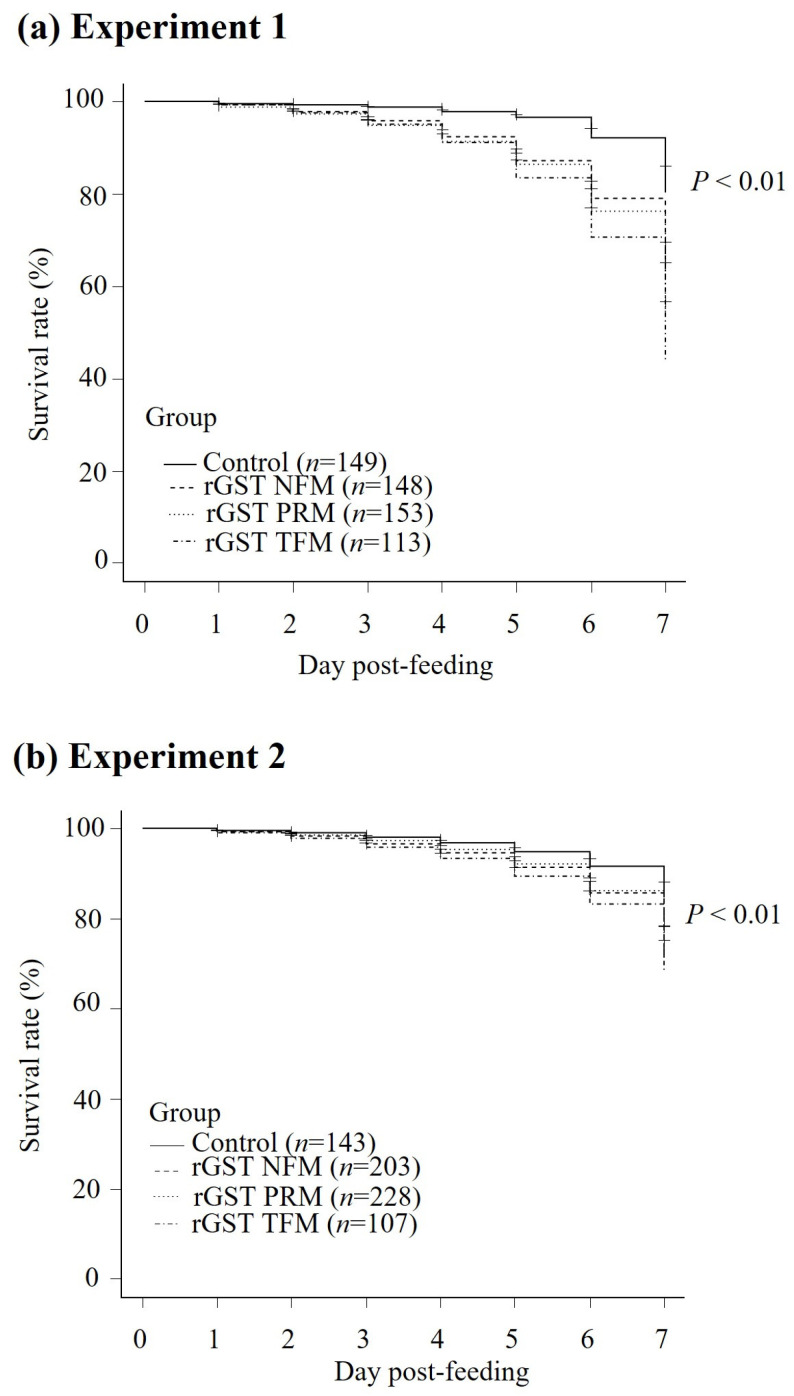
Evaluation of the acaricidal potential of GSTs by in vitro feeding with PRMs. The survival rate of PRMs fed with plasmas obtained from each immunized group were assessed daily for a week. Number of PRMs for these analyses are (**a**) Experiment 1: immune plasma: *n* = 153 (rGST PRM), *n* = 113 (rGST TFM), *n* = 148 (rGST NFM), control plasma: *n* = 149; (**b**) Experiment 2: immune plasma: *n* = 228 (rGST PRM), *n* = 107 (rGST TFM), *n* = 203 (rGST NFM), control plasma: *n* = 143. The number of dead PRMs was recorded. The statistical analysis was calculated by a log-rank test. *p* < 0.01 were considered statistically significant.

The potential of rGSTs for vaccine applications was investigated by feeding PRMs in vitro with fresh blood containing immune plasma against each rGST. We performed the in vitro feeding assays in duplicate. In Experiment 1, the mortality rate of PRMs gradually increased in each immunized group and reached 28.8, 38.9, and 23.6% in rGST PRM, rGST TFM, and rGST NFM, respectively, at 7 days post-feeding (Supplementary Table S3). In addition, the cumulative mortalities of PRMs at 2–7 days post-feeding in all immunized groups were significantly higher than those in the control group. A log-rank test and Kaplan–Meier analysis showed that the survival rate of PRMs significantly decreased after feeding with immune plasma against rGST TFM or rGST NFM, in addition to rGST PRM (Figure 6a). Likewise, Experiment 2 demonstrated that the survival rates of PRMs fed with plasma from each immunized group were significantly lower than those of the control group (Figure 6b). Significant differences in mortality rates were observed at 7 days post-feeding in the rGST PRM and rGST NFM immunized groups and at 5–7 days post-feeding in the rGST TFM immunized group (Supplementary Table S4). The cumulative mortality rate in the developmental stages of Experiment 1 was calculated (Supplementary Tables S5 and S6 and Supplementary Figure S3a,b) and significant differences were observed in both adults and nymphs. We did not analyze Experiment 2, because most of the PRMs used in this assay were nymphs and the sample distributions were not sufficient. These data revealed that immune plasma, including specific antibodies against each rGST, exhibited acaricidal effects on PRMs. Therefore, GST is potentially applicable as an antigen in the development of vaccines against avian mites.

## 4. Discussion

Avian mites, PRMs, TFMs, and NFMs, are debilitating pests on poultry farms that pose a threat to the poultry industry. Avian mites are distributed on poultry farms worldwide, although there are differences in mite species distributed in each region. Repeated long-term use of commonly used chemical compounds on farms has led to the emergence of acaricide-resistant mites, reducing the efficacy of chemicals and making the control of avian mites more difficult. Several vaccine antigens against PRMs have been reported as promising control strategies [21,22,23,24,25,26,27,28,29,30,31,32,33,34,35,36]. In addition, the application of homologous proteins present in different avian mites as vaccine antigens could be a valuable tool for the development of vaccines to control multiple avian mites. Recently, we reported that cysteine protease and ferritin 2 are highly conserved among avian mites, and that their application as vaccine antigens exhibits cross-protective immunity against avian mites [37,38]. In the present study, we characterized GSTs, which are important for the detoxification of acaricides in PRMs [54], and investigated their potential use as vaccine antigens against avian mites. We genetically characterized the GSTs from avian mites and examined their enzymatic activities. The immunogenicity of GSTs and cross-reactivity between each anti-GST plasma and recombinant GST (rGST) protein were assessed. In addition, we assessed the acaricidal efficacy of each anti-GST plasma by in vitro feeding assays using PRMs.

GSTs belong to a large superfamily of versatile proteins and are classified into various classes, such as cytosolic and mitochondrial GSTs, based on class-specific motifs and amino acid residues in the active sites involved in binding to their substrates [59]. Generally, GSTs are classified into alpha, mu, pi, theta, and sigma classes [60]. The GSTs identified in this study from avian mites belong to the mu class and are close to the same class of GSTs from other mite species. In common GST classes, the N-terminus shows a higher sequence similarity than the C-terminus [61,62]. The structure of GSTs of avian mites comprises two important domains: I at the N-terminus and II at the C-terminus, which are the general structures of GSTs of vertebrates and are required for their enzymatic function to detoxify and diminish the toxic effect of acaricides. In *Rhipicephalus microplus*, the active form of GSTs is a dimer, and each monomer contains eight helixes (α1–α8) and four beta-strands (β1–β4) [63]. In addition, GSTs, also known as allergens identified from *Dermatophagoides pteronyssinus* (house dust mite) and *Tyrophagus putrescentiae* mites, exhibit the dimer structure with eight helixes (α1–α8) and four beta-strands (β1–β4) [64]. In this study, we analyzed the alpha-fold monomeric structures of GSTs isolated from avian mites. The predicted monomer of each GST contained eight helixes (α1–α8), four beta-strands (β1–β4), two active sites (G- and H-sites), and mu loop, and these features were similar to the monomer GST of *R. microplus* and allergens of other mite genera [63,64], with their sequence homology ranging from 46–53%. In addition, the GSTs of avian mites showed a function in catalyzing the conjugation of GSH to CDNB, similar to those reported previously [54]. Moreover, mu-class GSTs in PRMs are involved in the detoxification of several classes of acaricides in the presence of GSH [54]. Therefore, the GSTs of avian mites identified in this study appear to be the cytosolic mu-class GST of vertebrates, which are necessary for catalytic activities and are involved in the detoxification of acaricides.

GST is considered as a vaccine candidate against many parasites in animals and humans. In hamsters and dogs, vaccination with recombinant *Necator americanus* GST (Na-GST-1) and *Ancylostoma caninum* GST-1 (*Ac*-GST-1), respectively, reduced worm burden and fecal egg counts [65,66]. The World Health Organization recommended GST as a vaccine antigen against *Schistosoma japonicum*, resulting in a reduced worm burden and number of eggs in experimental mice [67]. Thus, GST could be a promising vaccine antigen candidate. Moreover, the potential of homologous GST to induce cross-immunity has been reported, and the results of immunization with *Haemaphysalis longicornis* GST showed large differences in the cross-protective efficacy; immunization was 57% effective against *R. microplus* [52] and 67% effective against *R. appendiculatus,* but ineffective against *R. sanguineus* [48]. In addition, immunization with *Wuchereria bancrofti* rGST (rWbGST) induced higher levels of WbGST-specific IgG1 and IgG2a antibodies and pronounced IFN-γ, IL-10, and IL-4 cytokine production in vaccinated animals, while also demonstrating 65% in situ cytotoxicity to *Brugia malayi* (lymphatic filaria) [68]. Furthermore, the mRNA levels of delta- and mu-class GSTs were elevated in *Sarcoptes scabies* samples collected from a patient with recurrent crusted scabies over the course of their ivermectin treatment [69]. These observations strongly suggest the potential applications of GSTs in the development of universal vaccines. In the present study, we investigated the usefulness of GSTs from avian mites as vaccine antigens and confirmed that each rGST was immunogenic and that immunization produced high antibody titers in chickens. Moreover, the immune plasma from chickens immunized with each rGST cross-reacted with rGSTs from different mite species, and immune plasma against rGSTs from TFMs and NFMs exhibited acaricidal effects on PRMs. These results suggest that immunization with avian mite GSTs induces cross-immunity to protect against multiple avian mites. In this regard, immune plasma against rGSTs may trap the naive GSTs of mites, potentially inhibiting various mechanisms of metabolism and detoxification. To clarify whether the effect of vaccines against GSTs was induced by the inhibition of metabolism and detoxification in mites, the acaricidal effects of the vaccines on acaricide-resistant mites require further assessment. Moreover, we did not assess whether the cross-reactive antibodies in immune plasma can induce cytotoxicity across mite species. In addition, we need to consider that other immunization-induced factors may affect its acaricidal effects on PRMs. To precisely confirm the efficacy of these cross-reactive vaccine antigens, their acaricidal effects need to be examined using IgY purified from the immune plasma.

The mu-class GST is expressed in a variety of tissues and is involved in the detoxication of harmful organic compounds [70]. The GST family of proteins are conserved among various organisms and potentially share similar epitopes across species. According to previous reports, GST administration was not associated with autoimmune reactions [41], and in the phase 1 safety trial of Na-GST-1 immunization in humans, antigen-specific IgG antibodies were induced, and vaccine-related adverse effects were not observed [71]. However, the presence of long-lasting anti-GST antibodies in hosts may affect the crucial functions of GST. In addition to detoxification, some types of GSTs are known to be involved in various signaling cascades, including those involved in inflammation [72]. Therefore, the prolonged presence of anti-GST antibodies may make hosts more susceptible to infection. In this study, we did not observe any abnormalities in the health status of chickens during the experimental period; however, the long-term safety of GST immunization and its effects on immune responses need to be carefully assessed.

Recently, we reported the possible application of a single antigen as a universal vaccine for controlling avian mites [37,38]. This concept can encourage commercial production. In the present study, we assessed the potential application of GST as a broad-spectrum vaccine antigen against avian mites. Previously, GSTs were identified and characterized as molecules involved in acaricide detoxification in PRMs [44]. However, PRM GSTs have not been assessed as vaccine antigens. In this study, the immune plasma showed cross-reactivity with rGSTs from other mite species, and in vitro feeding assays revealed the acaricidal effects of immune plasma against the GSTs of TFMs/NFMs, in addition to PRMs. Furthermore, acaricidal effects were observed on both adult and nymph stages in Experiment 1. Thus, GSTs could be vaccine antigen candidates against PRMs; furthermore, they can be used as antigens in universal vaccines against avian mites. However, we assessed the acaricidal effects of immune plasma only on PRMs because of limited sample availability. To assess the potential of antigens for universal anti-tick vaccines, the efficacy of the vaccine antigens was assessed using in vitro feeding systems or by challenging immunized cattle with ticks [51,52,73]. Thus, the effects of an anti-PRM vaccine using PRM-GST as the vaccine antigen on TFMs/NFMs must be validated. However, in vitro feeding assays using TFMs/NFMs are not yet well established. Therefore, field trials challenging avian mites in immunized chickens may be an approach to assess the effects of anti-PRM vaccines on TFMs/NFMs. Although significant mortality rates of PRMs were observed in the immunized groups compared to the control groups, more than 70% of PRMs in immunized groups were alive after 7 days post-feeding. During the in vitro feeding in the present study, each PRM had an opportunity for blood feeding. Avian mites require blood feeding for development beyond the larval stage, and adult mites feed on blood up to 8 times during their egg-producing lifespan [74]. Therefore, multiple feedings of anti-GST containing blood may induce an additive effect on the survival of avian mites. In addition, to precisely evaluate the efficacies of vaccination with GSTs and effectively reduce the number of mites in poultry farms using vaccination approaches, we need to analyze the effects of these vaccines on the fecundity of adult mites fed with immune plasma and *GST* gene expression during different developmental stages. Future studies should also aim to identify suitable regions for the induction of antibodies that have highly acaricidal effects. Moreover, GSTs may enhance the acaricidal effects of other antigens (i.e., if included in a “cocktail vaccine”) because these organisms possess several antioxidant systems, including the thioredoxin system. Inhibition of drug metabolism and antioxidant activity by GST may facilitate the substitution of these systems with others [75], resulting in lowered acaricidal efficacy. Therefore, the identification of other common antigens and the combined use of multiple antigens could lead to the development of a cocktail vaccine with enhanced acaricidal effects against PRMs, TFMs, and NFMs.

## 5. Conclusions

In conclusion, we characterized the potential of GSTs from avian mites for the development of vaccines against avian mites. The GSTs of PRMs, TFMs, and NFMs are highly conserved among avian mites. In addition, rGSTs exhibit enzymatic activity in the conjugation of GSH to substrates, and their immunization successfully produces specific antibodies in chickens. Furthermore, immune plasma against rGST from PRMs, TFMs, and NFMs showed cross-reactivity with rGSTs from different avian mites and acaricidal effects on all avian mites. These results indicate the potential use of GSTs as vaccine antigens against avian mites, assuming that specific antibodies against GSTs inhibit the physiology of mites by reacting with naive GSTs in the midgut cells. Antigens common to mite species exhibiting similar biological characteristics represent their potential use as universal vaccine antigens. In addition, the combined use of other antigens as a cocktail vaccine may enhance the vaccine efficacy. Our study will serve as a basis for field trials challenging avian mites in immunized chickens to assess the effects of anti-PRM vaccines on TFMs/NFMs and clarify the effects of immunization with GST on acaricides in detail.

## Figures and Tables

**Table 1:** Sequence homology of glutathione S transferase among avian mites [poultry red mites (PRMs), tropical fowl mites (TFMs), and northern fowl mites (NFMs)].

	Amino Acid Sequence (%)			
		PRM	TFM	NFM
Nucleotide sequence (%)	PRM	-	80	80
	TFM	75	-	100
	NFM	75	100	-

**Table 2:** Antibody responses to recombinant glutathione S-transferase immunization.

Group	Chicken	Antibody Titer
Control	C1	<1000
	C2	<1000
	C3	<1000
	C4	<1000
Immunized-rGST PRM	PG1	32,000
	PG2	64,000
	PG3	64,000
	PG4	64,000
Immunized-rGST TFM	TG1	64,000
	TG2	64,000
	TG3	64,000
	TG4	64,000
Immunized-rGST NFM	NG1	64,000
	NG2	64,000
	NG3	64,000
	NG4	64,000

## Data Availability

The data presented in this study are available in the article and Supplementary Materials.

## Supplementary Materials

The following supporting information can be downloaded at: https://www.mdpi.com/article/10.3390/vaccines12020148/s1, Figure S1: The uncropped figure for Figure 3a,b; Figure S2: The uncropped figure for Figure 5; Figure S3; Evaluation of the acaricidal potential of GSTs by in vitro feeding with developmental stages of PRMs (Experiment 1); Table S1: List of primers used for the amplification of Glutathione S-transferase in this study; Table S2: List of Glutathione S-transferase genes used for phylogenetic analysis in Figure 2; Table S3: Mortality of mixed stages of PRMs fed with plasma from chickens immunized with glutathione S-transferase from different species of mites (Experiment 1); Table S4: Mortality of mixed stages of PRMs fed with plasma from chickens immunized with glutathione S-transferase from different species of mites (Experiment 2); Table S5: Mortality of adult PRMs fed with plasma from chickens immunized with glutathione S-transferase from different species of mites (Experiment 1); Table S6: Mortality of nymph PRMs fed with plasma from chickens immunized with glutathione S-transferase from different species of mites (Experiment 1).
