# Supplementary material for: In Vivo Characterization of the Anti-Glutathione S-Transferase Antibody Using an In Vitro Mite Feeding Model
Source: Vaccines (Basel). 2024 Jan 30;12(2):148. doi: 10.3390/vaccines12020148 (PMC10892040; doi:10.3390/vaccines12020148)
Supplement: Supplementary file 1 [file vaccines-12-00148-s001.zip › vaccines-2793418-supplementary.pdf]

Supplementary table S1. List of primers used for the amplification of Glutathione S-transferase in this study

Primer	Mite species	Intended use	Sequences (5´-3´)
GST outer -F	NFM & TFM	Partial gene amplification	TACTGGAACATCCGCGGMTT
GST outer- R			TGCCARTCGAGGAAYTCGWACA
GST inner -F			TTATCGAGAAGCAGGCYGATG
GST inner-R			AAGCGCTCRAGGAAKGCCTTKA
GSP1	NFM & TFM	3′RACE	AGCACAGCCCATTCGTAATCT
GSP2			TGGCGAGGAATATGAAGACCG
GSP3			AGCGATTGCCAACTACTGCT
GSP1	NFM & TFM	5′RACE	GAAGTCAACGTAGGTC
GSP2			AATCGCTTGCTTGAGGTCCA
GSP3			GGAAGATTGGGGAAGTCCAT
GST ORF-F	PRM, TFM & NFM	ORF	CGAGAGAAGTGGAGACAGCT
GST ORF-R			TGCTTGGAACGAGTTTGCTGA
rGST PRM-F	PRM	Construction for expression	AAGCATATGATGGCTCCGTCCACGACGTTC
rGST PRM-R		plasmids	ATCCTCGAGTCAGTTGCCCTTGGTCCAGCC
rGST TFM-F	NFM & TFM		AAGCATATGATGGCACCTTCAATTATTCTTGGTTACTGG
rGST TFM-R			ATCCTCGAGTCACTTATAGTAACCATATTTCTTGGCGAAGGG

*The NdeI and XhoI sites are underlined.

PRM, poultry red mite; NFM, northern fowl mite; TFM, tropical fowl mite; GST, Glutathione S-transferase; rGST, recombinant glutathione S-transferase

Supplementary Table S2 List of Glutathione S-transferase genes used for phylogenetic analysis in Figure 2

Accession No.	GenBank	Product	Organism
KR337505.1	*Dermanyssus gallinae* glutathione S-transferases-1 (GST-1)	glutathione S-transferases-1 (GST-1)	*Dermanyssus gallinae*
XM022854296.1	PREDICTED: *Varroa jacobsoni* glutathione S-transferase Mu 1-like (LOC111272717) mRNA	glutathione S-transferase Mu 1-like	*Varroa jacobsoni*
XM022792712.1	PREDICTED: *Varroa destructor* glutathione S-transferase Mu 1-like (LOC111245011) mRNA	glutathione S-transferase Mu 1-like	*Varroa destructor*
XM003747361.1	PREDICTED: *Metaseiulus occidentalis* glutathione S-transferase Mu 1 (LOC100904919) mRNA	glutathione S-transferase Mu 1	*Metaseiulus occidentalis*
KR337507.1	*Dermanyssus gallinae* glutathione S-transferases-3 (GST-3) mRNA complete cds	glutathione S-transferases-3 (GST-3)	*Dermanyssus gallinae*
XM029970261.3	PREDICTED: *Ixodes scapularis* glutathione S-transferase (LOC8032802) mRNA	glutathione S-transferase (LOC8032802) mRNA	*Ixodes scapularis*
XM037657656.2	PREDICTED: *Rhipicephalus sanguineus* glutathione S-transferase (LOC119390107) mRNA	glutathione S-transferase (LOC119390107) mRNA	*Rhipicephalus sanguineus*
XM037727032.2	PREDICTED: *Dermacentor silvarum* glutathione S-transferase-like (LOC119466514) mRNA	glutathione S-transferase-like (LOC119466514) mRNA	*Dermacentor silvarum*
XM037424660.1	PREDICTED: *Rhipicephalus microplus* glutathione S-transferase-like (LOC119173874) mRNA	glutathione S-transferase-like (LOC119173874) mRNA	*Rhipicephalus microplus*
XM037412779.1	PREDICTED: *Rhipicephalus microplus* glutathione S-transferase Mu 1-like (LOC119160027) mRNA	glutathione S-transferase Mu 1-like (LOC119160027) mRNA	*Rhipicephalus microplus*
MT665977.1	*Dermacentor marginatus* isolate XJ-Dm-09 Mu class glutathione S-transferase protein 4 (GSTM4) mRNA complete cds	glutathione S-transferase protein 4 (GSTM4) mRNA	*Dermacentor marginatus*
XM050192192.1	PREDICTED: *Dermacentor andersoni* glutathione S-transferase Mu 1-like (LOC126544729) transcript variant X1 mRNA	glutathione S-transferase Mu 1-like	*Dermacentor andersoni*

XM027349261.1	PREDICTED: *Dermatophagoides pteronyssinus* glutathione S-transferase D7-like (LOC113798691) mRNA	glutathione S-transferase D7-like	*Dermatophagoides pteronyssinus*
XM037722444.2	PREDICTED: *Dermacentor silvarum* glutathione S-transferase omega-1 (LOC119461218) mRNA	glutathione S-transferase omega-1	*Dermacentor silvarum*
NM001396288.1	*Gallus gallus* glutathione S-transferase theta 1 (GSTT1) transcript variant 2 mRNA	glutathione S-transferase theta 1 (GSTT1)	*Gallus gallus*
U13676.1	*Gallus gallus* liver theta glutathione-S-transferase mRNA complete cds	liver theta glutathione-S-transferase	*Gallus gallus*
MT665978.1	*Dermacentor marginatus* isolate XJ-Dm-12 Zeta class glutathione S-transferase protein 1 (GSTZ1) mRNA complete cds	Zeta class glutathione S-transferase protein 1 (GSTZ1) mRNA	*Dermacentor marginatus*
MT799206.1	*Diaphanosoma celebensis* GSTzeta protein mRNA complete cds	GSTzeta protein mRNA	*Diaphanosoma celebensis*
L15386.1	Chicken glutathione S-transferase (GST) mRNA complete cds	glutathione S-transferase (GST) mRNA	Chicken
NM204818.2	*Gallus gallus* glutathione S-transferase alpha 4 (GSTA4) mRNA	glutathione S-transferase alpha 4 (GSTA4) mRNA	*Gallus gallus*
MT665976.1	*Dermacentor marginatus* isolate XJ-Dm-03 Epsilon class glutathione S-transferase protein 1 (GSTE1) mRNA complete cds	Epsilon class glutathione S-transferase protein 1 (GSTE1)	*Dermacentor marginatus*
XM029978184.3	PREDICTED: *Ixodes scapularis* glutathione S-transferase 1-like (LOC8029517) mRNA	glutathione S-transferase 1-like	*Ixodes scapularis*
AB443867.1	*Culex quinquefasciatus* CqGSTd1 gene for glutathione transferase delta complete cds	glutathione transferase delta	*Culex quinquefasciatus*
JN251103.1	*Culex pipiens* glutathione S-transferase delta (GSTd1) mRNA complete cds	glutathione S-transferase delta (GSTd1) mRNA	*Culex pipiens*
KR337506.1	*Dermanyssus gallinae* glutathione S-transferases-2 (GST-2)	glutathione S-transferases-2 (GST-2)	*Dermanyssus gallinae*
GQ214698	*Sarcoptes scabiei* type suis glutathione S-transferase delta class 2 mRNA partial cds	glutathione S-transferase delta class 2	*Sarcoptes scabiei*

XM_027349261.1	PREDICTED: *Dermatophagoides pteronyssinus* glutathione S-transferase D7-like (LOC113798691) mRNA	glutathione S-transferase D7-like	*Dermatophagoides pteronyssinus*

Supplementary Table S3. Mortality of mixed stages of PRMs fed with plasma from chickens immunized with glutathione S-transferase from different species of mites (Experiment 1).

	Days post-feeding						
	**1**	**2**	**3**	**4**	**5**	**6**	**7**
**Control group (*n* = 149)**							
No. of dead PRMs post-feeding	3	3	3	6	6	14	19
Mortality (%)	2.0	2.0	2.0	4.0	4.0	9.4	12.8
**Immunized group (rGST PRM, *n* = 153)**							
No. of dead PRMs post-feeding	11	15	19	23	25	36	44
Mortality (%)	7.2	9.8	12.4	15.0	16.3	23.5	28.8
Chi-square	3.48	6.84	10.61	9.30	11.12	9.91	10.77
P value	0.052	6.01E-3*	5.67E-4*	1.43E-3*	4.78E-4*	1.06E-3*	6.6E-4*
Odds ratio	3.76	5.26	6.86	4.19	4.63	2.96	2.75
95% confidence interval	0.96–21.39	1.44—29.01	1.95–37.02	1.59–13.01	1.78–14.25	1.47–6.24	1.47–5.31
**Immunized group (rGST TFM, *n* = 113)**							
No. of dead PRMs post-feeding	6	11	15	18	29	35	44
Mortality (%)	5.3	9.7	13.3	15.9	25.7	30.9	38.9
Chi-square	1.17	5.97	10.79	9.29	23.63	17.74	22.01
P value	0.18	1.03E-2*	7.85E-4*	1.89E-3*	5.63E-7*	2.2E-5*	2.05E-6*
Odds ratio	2.68	5.13	7.27	4.41	8.01	4.22	4.24
95% confidence interval	0.55–16.89	1.31–29.36	1.98–40.21	1.60–14.08	3.10–24.58	2.06–9.04	2.23–8.33
**Immunized group (rGST NFM, *n* = 148)**							
No. of dead PRMs post-feeding	7	12	16	21	25	28	35
Mortality (%)	4.7	8.1	10.8	14.2	16.9	18.9	23.6
Chi-square	0.93	4.49	8.09	7.99	11.67	4.69	5.09
P value	0.335	0.0307*	3.32E-3*	3.95E-3*	4.5E-4*	2.94E-2*	2.34E-2*
Odds ratio	2.39	4.24	5.83	3.89	4.78	2.23	2.09
95% confidence interval	0.53–14.62	1.11–23.94	1.61–31.91	1.46–12.18	1.84–14.74	1.08–4.81	1.09–4.11

The mortality rate of PRMs was compared by Fisher's exact test between each immunized and control group.
*$P < 0.05$ was considered statistically significant.

Supplementary Table S4. Mortality of mixed stages of PRMs fed with plasma from chickens immunized with glutathione S-transferase from different species of mites (Experiment 2).

	Days post-feeding						
	**1**	**2**	**3**	**4**	**5**	**6**	**7**
**Control group (*n* =143)**							
No. of dead PRMs post-feeding	4	5	6	8	8	10	11
Mortality (%)	2.8	3.5	4.2	5.6	5.6	6.9	7.7
**Immunized group (rGST PRM, *n*=228)**							
No. of dead PRMs post-feeding	9	11	15	18	23	29	41
Mortality (%)	3.9	4.8	6.6	7.9	10.1	12.7	17.9
Chi-square	0.08	0.48	0.54	0.40	1.76	2.48	6.89
P value	0.773	0.423	0.368	0.531	0.176	0.0849	5.49E-3*
Odds ratio	1.42	1.75	1.60	1.44	1.89	1.93	2.62
95% confidence interval	0.38–6.46	0.51–7.72	0.57–5.17	0.58–3.95	0.78–5.03	0.88–4.60	1.26–5.87
**Immunized group (rGST TFM, *n*=107)**							
No. of dead PRMs post-feeding	6	9	10	11	14	15	20
Mortality (%)	5.6	8.4	9.3	10.2	13.3	14.0	18.7
Chi-square	0.63	1.94	1.91	1.30	3.39	2.62	5.84
P value	0.334	0.104	0.12	0.228	0.0443*	0.0877	0.0114*
Odds ratio	2.05	2.52	2.34	1.92	2.53	2.16	2.74
95% confidence interval	0.47–10.18	0.73–9.89	0.74–8.12	0.67–5.74	0.94–7.25	0.86–5.63	1.18–6.68
**Immunized group (rGST NFM, *n*=203)**							
No. of dead PRMs post-feeding	9	12	17	18	21	25	35
Mortality (%)	4.4	5.9	8.4	8.9	10.3	12.3	17.2
Chi-square	0.25	0.59	1.73	0.86	1.88	2.06	5.83
P value	0.57	0.45	0.187	0.304	0.167	0.147	0.0101*
Odds ratio	1.61	1.73	2.08	1.63	1.94	1.86	2.49
95% confidence interval	0.43–7.29	0.55–6.42	0.75–6.62	0.65–4.49	0.79–5.23	0.83–4.50	1.18–5.65

The mortality rate of PRMs was compared by Fisher's exact test between each immunized and control group.
*$P < 0.05$ was considered statistically significant.

Supplementary Table S5. Mortality of adult PRMs fed with plasma from chickens immunized with glutathione S-transferase from different species of mites (Experiment 1)

	Days post-feeding						
	**1**	**2**	**3**	**4**	**5**	**6**	**7**
**Control group (*n* =50)**							
No. of dead PRMs post-feeding	2	2	2	4	4	8	11
Mortality (%)	4	4	4	8	8	16	22
**Immunized group (rGST PRM, *n*=76)**							
No. of dead PRMs post-feeding	7	11	14	15	16	19	23
Mortality (%)	9.2	14.4	18.4	19.7	21.1	25	30.3
Chi-square	0.573	2.5331	4.431	2.3925	2.9324	0.9656	0.66783
P value	0.316	0.0748	0.0262*	0.0807	0.0792	0.272	0.412
Odds ratio	2.419	4.024	5.361	2.806	3.042	1.74	1.53
95% confidence interval	0.43-24.85	0.82-39.03	1.14-50.84	0.82-12.39	0.89-13.35	0.65-5.06	0.63-3.92
**Immunized group (rGST TFM, *n*=58)**							
No. of dead PRMs post-feeding	3	6	10	10	11	15	23
Mortality (%)	5.2	10.3	17.2	17.2	18.9	25.9	39.7
Chi-square	1.23E-31	0.78672	3.5205	1.296	1.8606	1.0253	3.1049
P value	1	0.282	0.0342*	0.25	0.162	0.245	0.0622
Odds ratio	1.305	2.745	4.934	2.377	0.723	1.821	2.311
95% confidence interval	0.14-16.24	0.46-29.09	0.97-48.67	0.63-11.13	0.72-12.33	0.64-5.52	0.92-6.06
**Immunized group (rGST NFM, *n*=69)**							
No. of dead PRMs post-feeding	3	8	12	14	15	16	18
Mortality (%)	4.3	11.6	17.4	20.3	21.7	23.2	26.1
Chi-square	1.62E-31	1.2976	3.8014	2.5207	3.119	0.53755	0.087781
P value	1	0.189	0.0406*	0.0745	0.0472*	0.365	0.669
Odds ratio	1.09	3.1209	4.995	2.903	3.166	1.578	1.249
95% confidence interval	0.12-13.52	0.58-31.49	1.03-48.14	0.83-12.95	0.92-14.03	0.57-4.69	0.49-3.29

The mortality rate of PRMs was compared by Fisher's exact test between each immunized and control group.
*P < 0.05 was considered statistically significant.

Supplementary Table S6. Mortality of nymph PRMs fed with plasma from chickens immunized with glutathione S-transferase from different species of mites (Experiment 1)

	Days post-feeding						
	**1**	**2**	**3**	**4**	**5**	**6**	**7**
**Control group (*n* =99)**							
No. of dead PRMs post-feeding	1	1	1	2	2	6	8
Mortality (%)	1	1	1	2	2	6.1	8.1
**Immunized group (rGST PRM, *n*=77)**							
No. of dead PRMs post-feeding	4	4	5	8	9	17	21
Mortality (%)	5.2	5.2	6.5	10.4	11.7	22.1	27.3
Chi-square	1.4409	1.4409	2.465	4.2073	5.358	8.4222	10.239
P value	0.17	0.17	0.0875	0.0222*	0.0112*	0.00276*	8.92E-4*
Odds ratio	5.323	5.323	6.739	5.571	6.357	4.354	4.229
95% confidence interval	0.51-266.7	0.51-266.71	0.73-324.55	1.06-55.4	1.25-62.32	1.53-14.27	1.66-11.82
**Immunized group (rGST TFM, *n*=55)**							
No. of dead PRMs post-feeding	3	5	5	8	18	20	21
Mortality (%)	5.5	9.1	9.1	14.5	32.7	36.4	38.2
Chi-square	1.2833	4.1968	4.1968	7.189	26.848	21.028	19.036
P value	0.13	0.0222*	0.0222*	0.00428*	1.14E-7*	5.78E-06*	9.4E-06*
Odds ratio	5.588	9.658	9.658	8.138	23.095	8.712	6.922
95% confidence interval	0.43-299.24	1.04-466.93	1.04-466.93	1.54-81.82	5.13-214.21	3.05-28.78	2.64-19.88
**Immunized group (rGST NFM, *n*=79)**							
No. of dead PRMs post-feeding	4	4	4	7	10	12	17
Mortality (%)	5.1	5.1	5.1	8.9	12.7	15.2	21.5
Chi-square	1.3678	1.3678	1.3678	2.9764	6.3073	3.0869	5.5065
P value	0.173	0.173	0.173	0.0798	0.00613*	0.0774	0.0158*
Odds ratio	5.183	5.183	5.183	4.676	6.96	2.76	3.098
95% confidence interval	0.49-259.61	0.49-259.61	0.49-259.61	0.85-47.43	1.42-67.39	0.90-9.43	1.18-8.83

The mortality rate of PRMs was compared by Fisher’s exact test between each immunized and control group.
*$P < 0.05$ was considered statistically significant.
