# Supplementary material for: In Vivo Characterization of the Anti-Glutathione S-Transferase Antibody Using an In Vitro Mite Feeding Model
Source: Vaccines (Basel). 2024 Jan 30;12(2):148. doi: 10.3390/vaccines12020148 (PMC10892040; doi:10.3390/vaccines12020148)
Supplement: Supplementary file 1 [file vaccines-12-00148-s001.zip › vaccines-2793418-supplementary.pptx]

## Slide 1
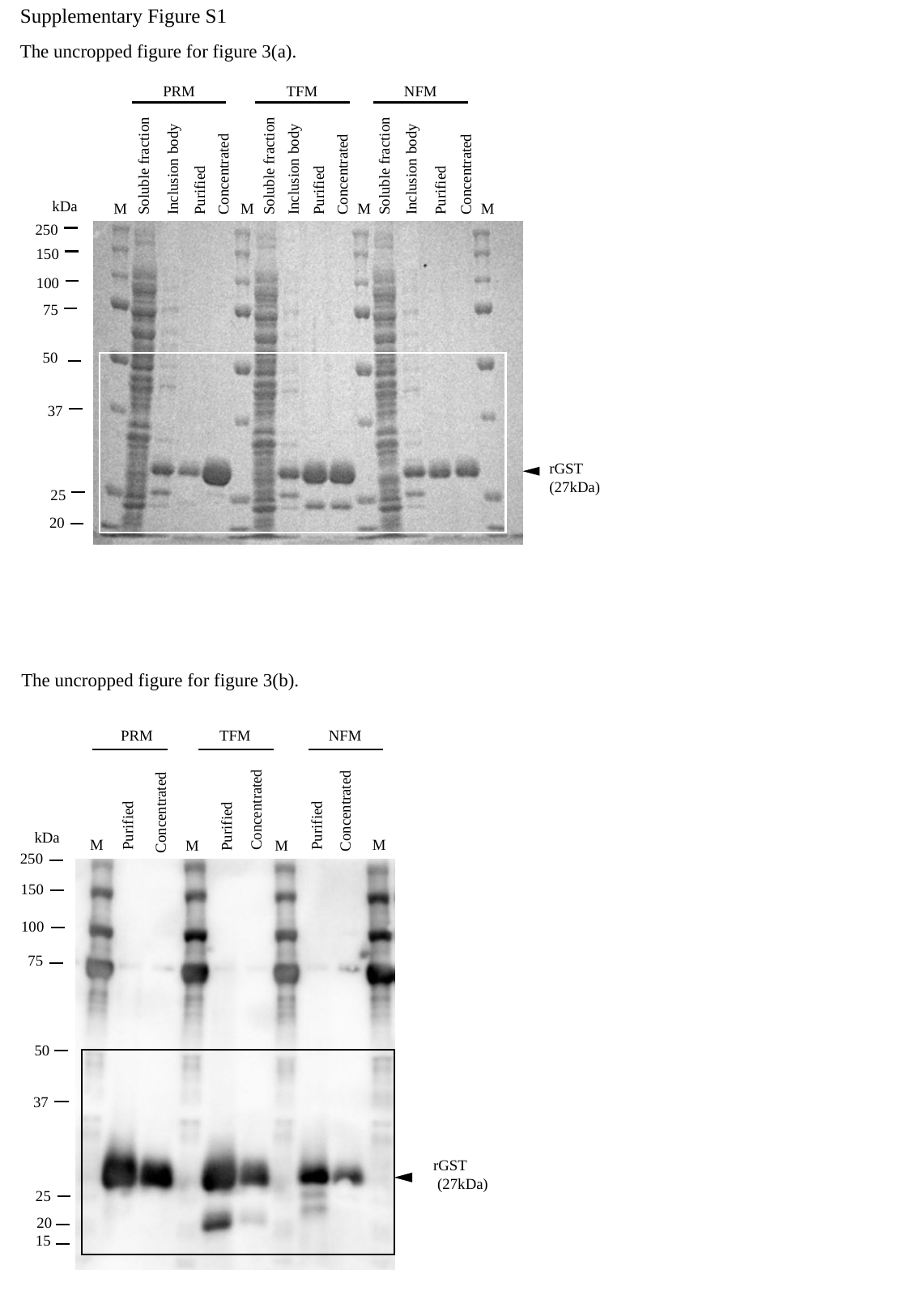

Supplementary Figure S1
The uncropped figure for figure 3(a).
PRM
TFM
NFM
Soluble fraction
Soluble fraction
Soluble fraction
Inclusion body
Inclusion body
Inclusion body
Purified
Concentrated
Concentrated
Concentrated
Purified
Purified
M
M
M
M
rGST (27kDa)
kDa
250
150
100
75
50
37
25
20
The uncropped figure for figure 3(b).
PRM
TFM
NFM
Concentrated
Purified
Purified
Concentrated
Purified
Concentrated
M
M
M
M
rGST
 (27kDa)
kDa
250
150
100
75
50
37
25
20
15

## Slide 2
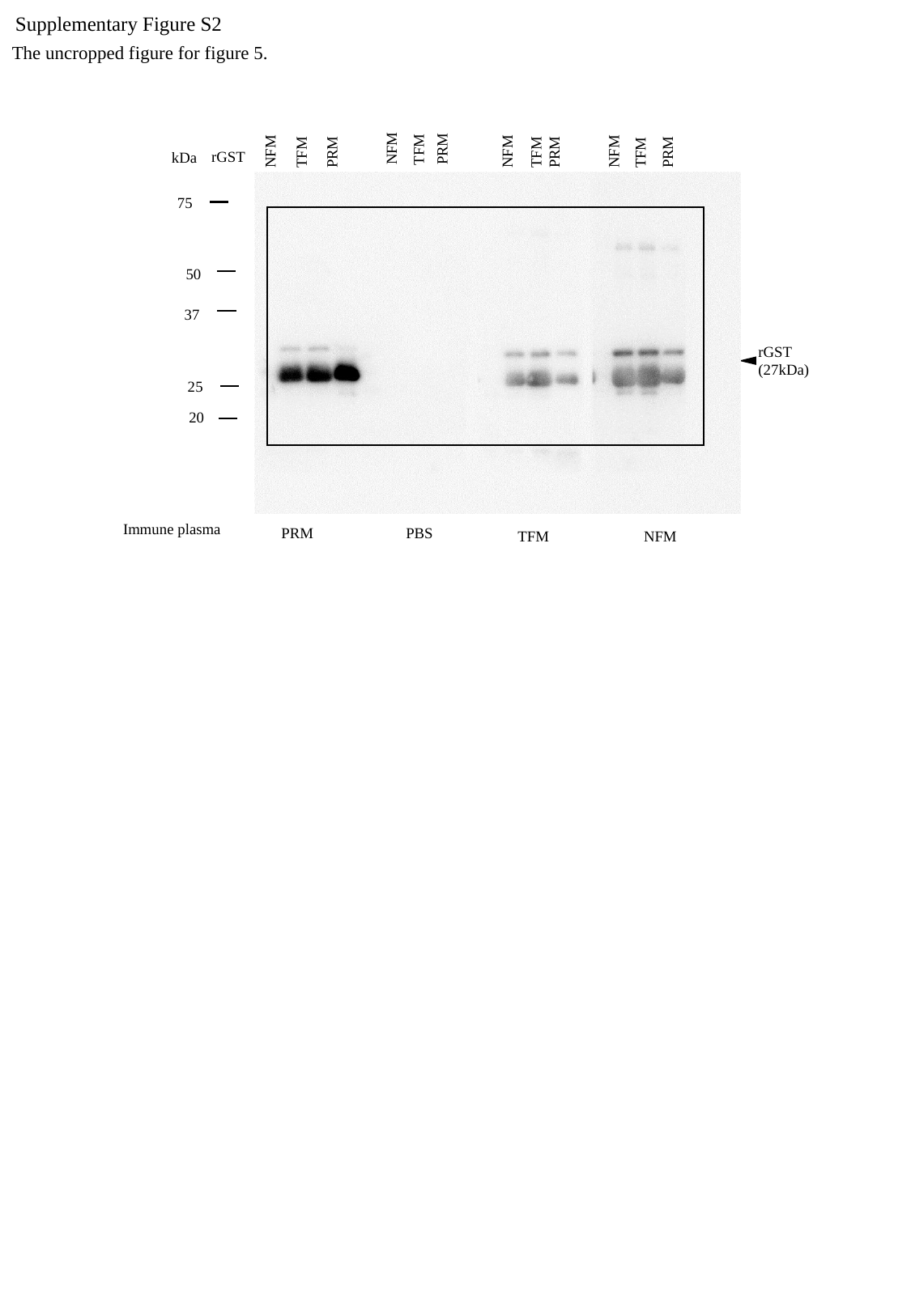

Supplementary Figure S2
The uncropped figure for figure 5.
NFM
PRM
PRM
NFM
NFM
PRM
PRM
NFM
TFM
TFM
TFM
TFM
rGST
rGST
(27 kDa)
Immune plasma
PRM
PBS
TFM
NFM
rGST (27kDa)
kDa
75
50
37
25
20

## Slide 3
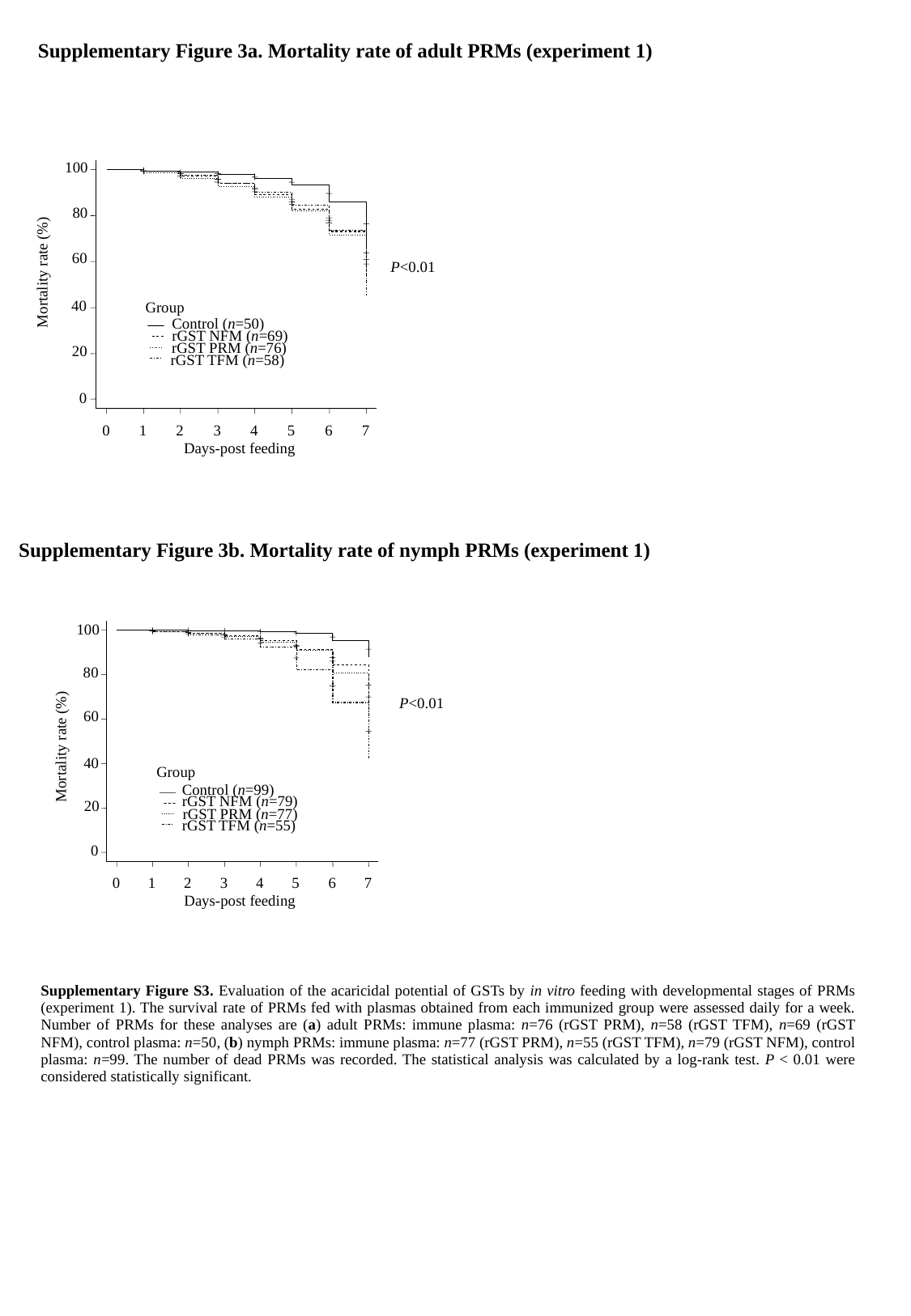

Supplementary Figure 3a. Mortality rate of adult PRMs (experiment 1)
100
80
60
Mortality rate (%)
40
Group
Control (n=50)
rGST NFM (n=69)
rGST PRM (n=76)
20
rGST TFM (n=58)
0
0
1
2
3
4
5
6
7
Days-post feeding
P<0.01
Supplementary Figure 3b. Mortality rate of nymph PRMs (experiment 1)
100
80
60
Mortality rate (%)
40
Group
Control (n=99)
rGST NFM (n=79)
20
rGST PRM (n=77)
rGST TFM (n=55)
0
0
1
2
3
4
5
6
7
Days-post feeding
P<0.01
Supplementary Figure S3. Evaluation of the acaricidal potential of GSTs by in vitro feeding with developmental stages of PRMs (experiment 1). The survival rate of PRMs fed with plasmas obtained from each immunized group were assessed daily for a week. Number of PRMs for these analyses are (a) adult PRMs: immune plasma: n=76 (rGST PRM), n=58 (rGST TFM), n=69 (rGST NFM), control plasma: n=50, (b) nymph PRMs: immune plasma: n=77 (rGST PRM), n=55 (rGST TFM), n=79 (rGST NFM), control plasma: n=99. The number of dead PRMs was recorded. The statistical analysis was calculated by a log-rank test. P < 0.01 were considered statistically significant.
